# The soil‐borne ultimatum, microbial biotechnology and sustainable agriculture

**DOI:** 10.1111/1751-7915.13947

**Published:** 2021-10-10

**Authors:** Peter A. H. M. Bakker, Roeland L. Berendsen

**Affiliations:** ^1^ Plant‐Microbe Interactions Department of Biology Utrecht University Padualaan 8 Utrecht 3584 CH the Netherlands

## The root microbiome and plant growth

The human population is increasing at a staggering rate and will require strongly increased agricultural production to feed it (FAO, 2009). This situation forces us to critically explore the resources needed to produce crops in a durable way. The main resource for agriculture is the soil in which crops are produced. Soil is a source of essential nutrients for the plant, and soil microbes play a key role in making such nutrients available (Oldroy and Leyser, 2020). Moreover, soil microbes are also essential in protecting plants against abiotic and biotic stresses (Jones *et al*., 2019). In this context, soil health is defined as the capacity of a living soil to sustain plant productivity and to promote plant health (Doran, 2002). The microbial communities associated with the plant are now considered to function as the extended genotype of the plant and are generally referred to as the plant microbiome (Turner *et al*., 2013). We therefore believe that the adage ‘healthy roots, healthy plants, healthy people’ (Dr. David Weller, Washington state University) needs to be preceded by ‘healthy soils’, as the essential prerequisite to produce healthy plants.

However, the soil not only contains microbes that sustain plant growth but also those that are detrimental to the plant (Mendes *et al*., 2013). Thus, a delicate balance in the complex plant associated microbial communities is required to sustain plant vigour (Berendsen *et al*., 2012). Obviously, pathogenic microbes, including bacteria, fungi and oomycetes, can negatively affect plant growth (Agrios, 2005). Soil‐borne plant pathogens can accumulate in the soil and reduce health of crops over the years (Bennett *et al*., 2012). Also microbes in general can negatively affect plant performance as most microbes produce the so‐called microbe‐associated molecular patterns (MAMPs) that can activate root immune responses resulting in root growth inhibition (Yu *et al*., 2019b). As the diverse microbiota present in the root associated microbiome, all have such conserved MAMPs, for example, bacterial flagellin and fungal chitin (Pel and Pieterse, 2013), the root environment is rich in these potentially plant growth inhibiting molecules, and it is actually remarkable that plants can grow in microbially active soils. Recent findings suggest that microbes not only trigger root immune responses but many of the microbiota also actively suppress these responses (Yu *et al*., 2019a; Ma *et al*., 2021). For plant beneficial *Pseudomonas* bacteria, suppression of MAMP‐triggered root immune responses is mediated by simply lowering environmental pH (Yu *et al*., 2019a). Further elucidation of the molecular communication that underlies the balance between detrimental and beneficial effects of the root associated microbiome on plant growth is urgently needed for applications in agricultural settings.

## The soil‐borne legacy

There are examples of soils in which soil‐borne pathogens are effectively controlled by specific members of the root microbiome, the so‐called disease suppressive soils (Schlatter *et al*., 2017). In such soils, a disease outbreak is required to enrich specific disease‐suppressive microbiota in the rhizosphere, resulting in a suppressive soil memory that is activated by new infections (Raaijmakers and Mazzola, 2016). Exploitation of this phenomenon in agriculture can be achieved by either isolating and introducing the disease suppressive microbes or by engineering the indigenous microbiome by practices that sustain the populations and activities of such microbes. Again identifying key signalling compounds involved in beneficial interactions between the root microbiome and the plant is essential. For interactions that govern suppression of soil‐borne diseases, the microbiome is influenced by both the pathogen and the plant, making it difficult to distinguish signals from the plant, the pathogen and the pathogenic interaction. The aboveground parts of plants are also attacked by pathogens and pests, and recent evidence suggests that such interactions also result in effects on the root microbiome (Kong *et al*., 2016; Berendsen *et al*., 2018; Yuan *et al*., 2018). Whitefly infestation of *Capsicum annuum* (pepper) leaves reshaped the root‐associated microbiome, and it was shown that fluorescent pseudomonads that are recruited after the insect infestation have insect‐killing capacity (Kong *et al*., 2016). Infection of *Arabidopsis thaliana* (thale cress) leaves with the oomycete *Hyaloperonospora arabidopsidis* caused an enrichment of the root microbiome with a consortium of three bacterial species (Berendsen *et al*., 2018). When a new generation of *A. thaliana* was grown on soil conditioned with pathogen infected plants, they were significantly less susceptible to *H. arabidopsidis* infection than plants grown on soil in which healthy *A. thaliana* were previously grown. Moreover, when the consortium of the three bacterial species was isolated and introduced into soil, they protected *A. thaliana* against infection with the oomycete pathogen (Berendsen *et al*., 2018). Likewise, infection of *A. thaliana* leaves with the pathogenic bacterium *Pseudomonas syringae* pv. *tomato* resulted in shifts in the root‐associated microbiome, accompanied by a protective effect against *P. syringae* pv. *tomato* in subsequent *A. thaliana* generations (Yuan *et al*., 2018). Thus, it seems that plants can recruit specific microbiota under stress conditions that are in turn effective in protecting a future generation of plants against the stress condition, a phenomenon dubbed the soil‐borne legacy (Bakker *et al*., 2018). The protective effect of the recruited microbiome could well be induced systemic resistance (ISR), where microbes on the root trigger a systemic response in the plant that leads to a primed state in which plants mount defence responses much faster and to a greater magnitude after pathogen infection (Conrath *et al*., 2002). Indeed, many beneficial microbes have been demonstrated to elicit ISR when they colonize plant roots (Pieterse *et al*., 2014), however, experimental evidence for the actual involvement of ISR in soil‐borne legacies has as yet not been reported. More than 30 years ago, ISR was discovered to be a potent mode of action of beneficial microbes to control plant diseases (Van Peer *et al*., 1991; Wei *et al*., 1991), yet application of ISR‐eliciting microbes in agricultural practice is not fully developed, due to limited and unpredictable efficacy. Determining predictors of ISR efficacy under different environmental conditions is therefore an essential development to expand the use of ISR‐eliciting microbes in sustainable agriculture (Lee Diaz *et al*., 2021). Both for the recruitment and the efficacy of ISR‐eliciting microbes, fine‐tuned molecular signalling seems to be required, and dissecting signalling may be key in optimizing applications.

## Signals from the underground

Microbial elicitors of ISR include lipopolysaccharides, flagella, siderophores, antibiotics, N‐acyl homoserine lactones, biosurfactants and a range of volatile compounds (Pieterse *et al*., 2014, 2021). Because such inducing molecules are widespread in the root microbiome, it seems most likely that actual production by the microbes and perception by the plant are important factors in determining the efficacy of ISR. Moreover, we are beginning to understand the molecular mechanisms and plant signals that affect recruitment and biological control activity of beneficial microbes (Song *et al*., 2021), opening possibilities to develop plant breeding programs that include microbiome functioning (Pieterse *et al*., 2016). A class of plant metabolites that has attracted recent attention in relation to plant microbe interactions are the coumarins (Stringlis *et al*., 2019). They have been identified as important semiochemicals in plant–microbe interactions and in assembly of the root microbiome (Stringlis *et al*., 2018; Voges *et al*., 2019; Harbort *et al*., 2020). Coumarins have been reported to accumulate in plants under pathogen attack, and they display selective antimicrobial activity (Stringlis *et al*., 2019). In *A. thaliana*, the root microbiomes of wild‐type plants and a mutant defective in the production and root excretion of the coumarin scopoletin displayed clear differences in which some microbial genera were enriched on the wild‐type roots, whereas others were enriched on the roots of mutant plants (Stringlis *et al*., 2018). Moreover, in this study, the *in vitro* growth of a selection of plant beneficial *Pseudomonas* bacteria was insensitive to scopoletin, whereas that of several plant pathogenic fungi was strongly inhibited. Thus, coumarins seem to specifically stimulate or inhibit microbiota on plant roots. Coumarins not only affect microbiome assembly but also seem to influence activity of microbes. The transcriptome of a plant beneficial *Pseudomonas simiae* was significantly affected by coumarins in root exudates of *A. thaliana* (Yu *et al*., 2021). In this study, genes related to transport and metabolism of range of compounds were upregulated by coumarins and some genes, for example, motility‐related genes, were downregulated. Thus, the pathogen inhibitory activity of beneficial microbes on plant roots may be fine‐tuned by the plant under specific conditions. An intriguing role of coumarins in evolution of plant root inhabiting *Pseudomonas protegens* strain CHA0 was recently reported by Li *et al*. (2021). In their experimental evolution study, strain CHA0 was grown on the roots of gnotobiotic *A. thaliana*. After 4 weeks of plant growth, the bacterial population was transferred to fresh gnotobiotic plants, and this was repeated several times. The bacteria evolved into plant growth‐stimulating mutualists, and these mutualists induced enhanced expression of *MYB72*, a gene encoding a transcription factor regulating scopoletin production; moreover, the evolved mutualists were less sensitive to the antimicrobial activity of scopoletin (Li *et al*., 2021). Thus, coumarins may play an important role in domestication of bacteria that inhabit plant roots. Whether such coumarin‐regulated communication is also effective in complex root‐associated microbiomes remains to be investigated. Identification of signals from the underground that orchestrate assembly and functioning of microbiomes will stimulate applications of this knowledge in agriculture. We think that applications will include the addition of semiochemicals to selectively stimulate resident beneficial microbes or to use them as supplements to inoculum of beneficial microbes.

## Concluding remarks

The essential role of soil microbes in healthy roots and healthy plants was postulated more than a century ago by Lorentz Hiltner (1904), and his point of view is still valid and vibrant (Bakker *et al*., 2020). Recently, the well‐known phenomenon that hybrid cultivars of crops grow better than their inbred parent lines was surprisingly also linked to soil microbes (Wagner *et al*., 2021). The soil‐borne ultimatum to optimize plant growth and health by managing the soil microbiome may now be facilitated by the rapid developments in high‐throughput analyses of microbiome composition and activities. Detailed insights into the chemical communication between plant roots and their associated microbiome will offer opportunities for sensible interference to increase crop productivity. For broad‐scale application, the acceptance of microbiome‐assisted agriculture by the public is an important issue (Thomashow *et al*., 2019) and should be included in discussions between farmers, scientists, industry and legislators. In the past 15 years, we have witnessed an unprecedented increase in our knowledge of the soil microbiome; we can hardly wait to look back 15 years from now to see how microbial biotechnology has influenced our agricultural food production.

## References

[mbt213947-bib-0001] Agrios, G.N. (2005) Plant Pathology, 5th edn. Cambridge, MA: Elsevier Academic Press.

[mbt213947-bib-0002] Bakker, P.A.H.M. , Berendsen, R.L. , Van Pelt, J.A. , Vismans, G. , Yu, K. , Li, E.Q. , *et al*. (2020) The soil‐borne identity and microbiome‐assisted agriculture: looking back to the future. Mol Plant 13: 1394–1401. 32979564 10.1016/j.molp.2020.09.017

[mbt213947-bib-0003] Bakker, P.A.H.M. , Pieterse, C.M.J. , De Jonge, R. , and Berendsen, R.L. (2018) The soil‐borne legacy. Cell 172: 1178–1180. 29522740 10.1016/j.cell.2018.02.024

[mbt213947-bib-0004] Bennett, A.J. , Bending, G.D. , Chandler, D. , Hilton, S. , and Mills, P. (2012) Meeting the demand for crop production: the challenge of yield decline in crops grown in short rotations. Biol Rev 87: 52–71. 21631700 10.1111/j.1469-185X.2011.00184.x

[mbt213947-bib-0005] Berendsen, R.L. , Pieterse, C.M.J. , and Bakker, P.A.H.M. (2012) The rhizosphere microbiome and plant health. Trends Plant Sci 17: 478–486. 22564542 10.1016/j.tplants.2012.04.001

[mbt213947-bib-0006] Berendsen, R.L. , Vismans, G. , Yu, K. , Song, Y. , Burgman, W. , Burmølle, M. , *et al*. (2018) Disease‐induced assemblage of a plant‐beneficial bacterial consortium. ISME J 12: 1496–1507. 29520025 10.1038/s41396-018-0093-1 PMC5956071

[mbt213947-bib-0007] Conrath, U. , Pieterse, C.M.J. , and Mauch‐Mani, B. (2002) Priming in plant‐pathogen interactions. Trends Plant Sci 7: 210–216. 11992826 10.1016/s1360-1385(02)02244-6

[mbt213947-bib-0008] Doran, J.W. (2002) Soil health and global sustainability: translating science into practice. Agric Ecosyst Environ 88: 119–127.

[mbt213947-bib-0009] FAO . (2009) Feeding the World in 2050. World Agricultural Summit on Food Security. Rome, Italy: FAO.

[mbt213947-bib-0010] Harbort, C.J. , Hashimoto, M. , Inoue, H. , Niu, Y. , Guan, R. , Rombolà, A.D. , *et al*. (2020) Root‐secreted coumarins and the microbiota interact to improve iron nutrition in arabidopsis. Cell Host Microbe 28: 825–837. 33027611 10.1016/j.chom.2020.09.006 PMC7738756

[mbt213947-bib-0011] Hiltner, L. (1904) Uber neuere Erfahrungen und Probleme auf dem Gebiete der Bodenbakteriologie unter besonderer Berucksichtigung der Grundungung und Brache. Arb. DLG 98: 59–78.

[mbt213947-bib-0012] Jones, P. , Garcia, B.J. , Furches, A. , Tuskan, G.A. , and Jacobson, D. (2019) Plant host‐associated mechanisms for microbial selection. Front Plant Sci 10: 862. 31333701 10.3389/fpls.2019.00862 PMC6618679

[mbt213947-bib-0013] Kong, H.G. , Kim, B.K. , Song, G.C. , Lee, S. , and Ryu, C.M. (2016) Aboveground whitefly infestation‐mediated reshaping of the root microbiota. Front Microbiol 7: 1314. 27656163 10.3389/fmicb.2016.01314 PMC5013075

[mbt213947-bib-0014] Lee Díaz, A.S. , Macheda, D. , Saha, H. , Ploll, U. , Orine, D. , and Biere, A. (2021) Tackling the context‐dependency of microbial‐induced resistance. Agronomy 11: 1293.

[mbt213947-bib-0015] Li, E.Q. , De Jonge, R. , Liu, C. , Jiang, H.N. , Friman, V.P. , Pieterse, C.M.J. , *et al*. (2021) Rapid evolution of bacterial mutualism in the plant rhizosphere. Nat Commun 12: 3829. 34158504 10.1038/s41467-021-24005-y PMC8219802

[mbt213947-bib-0016] Ma, K.W. , Niu, Y. , Jia, Y. , Ordon, J. , Copeland, C. , Emonet, A. , *et al*. (2021) Coordination of microbe–host homeostasis by crosstalk with plant innate immunity. Nat Plants 7: 814–825. 34031541 10.1038/s41477-021-00920-2 PMC8208891

[mbt213947-bib-0017] Mendes, R. , Garbeva, P. , and Raaijmakers, J.M. (2013) The rhizosphere microbiome: significance of plant beneficial, plant pathogenic, and human pathogenic microorganisms. FEMS Microbiol Rev 37: 634–666. 23790204 10.1111/1574-6976.12028

[mbt213947-bib-0018] Oldroy, G.E.D. , and Leyser, O. (2020) A plant’s diet, surviving in a variable nutrient environment. Science 368: eaba0196. 32241923 10.1126/science.aba0196

[mbt213947-bib-0019] Pel, M.J.C. , and Pieterse, C.M.J. (2013) Microbial recognition and evasion of host immunity. J Exp Bot 64: 1237–1248. 23095994 10.1093/jxb/ers262

[mbt213947-bib-0020] Pieterse, C.M.J. , Berendsen, R.L. , de Jonge, R. , Stringlis, I.A. , Van Dijken, A.J.H. , Van Pelt, J.A. , *et al*. (2021) *Pseudomonas* *simiae* WCS417: star track of a model beneficial rhizobacterium. Plant Soil 461: 245–263.

[mbt213947-bib-0021] Pieterse, C.M.J. , De Jonge, R. , and Berendsen, R.L. (2016) The soil‐borne supremacy. Trends Plant Sci 21: 171–173. 26853594 10.1016/j.tplants.2016.01.018

[mbt213947-bib-0022] Pieterse, C.M.J. , Zamioudis, C. , Berendsen, R.L. , Weller, D.M. , Van Wees, S.C.M. , and Bakker, P.A.H.M. (2014) Induced systemic resistance by beneficial microbes. Annu Rev Phytopathol 52: 347–375. 24906124 10.1146/annurev-phyto-082712-102340

[mbt213947-bib-0023] Raaijmakers, J.M. , and Mazzola, M. (2016) Soil immune responses. Science 352: 1392–1393. 27313024 10.1126/science.aaf3252

[mbt213947-bib-0024] Schlatter, D. , Kinkel, L. , Thomashow, L. , Weller, D. , and Paulitz, T. (2017) Disease suppressive soils: new insights from the soil microbiome. Phytopathology 107: 1284–1297. 28650266 10.1094/PHYTO-03-17-0111-RVW

[mbt213947-bib-0025] Song, Y. , Wilson, A.J. , Zhang, X.C. , Thoms, D. , Sohrabi, R. , Song, S. , *et al*. (2021) FERONIA restricts *Pseudomonas* in the rhizosphere microbiome via regulation of reactive oxygen species. Nat Plants 7: 644–654. 33972713 10.1038/s41477-021-00914-0

[mbt213947-bib-0026] Stringlis, I.A. , De Jonge, R. , and Pieterse, C.M.J. (2019) The age of coumarins in plant–microbe interactions. Plant Cell Physiol 60: 1405–1419. 31076771 10.1093/pcp/pcz076 PMC6915228

[mbt213947-bib-0027] Stringlis, I.A. , Yu, K. , Feussner, K. , De Jonge, R. , Van Bentum, S. , Van Verk, M.C. , *et al*. (2018) MYB72‐dependent coumarin exudation shapes root microbiome assembly to promote plant health. Proc Natl Acad Sci USA 115: 5213–5222. 10.1073/pnas.1722335115 PMC5984513 29686086

[mbt213947-bib-0028] Thomashow, L.S. , LeTourneau, M.K. , Kwak, Y.S. , and Weller, D.M. (2019) The soil‐borne legacy in the age of the holobiont. Microb Biotechnol 12: 51–54. 30328280 10.1111/1751-7915.13325 PMC6302707

[mbt213947-bib-0029] Turner, T.R. , James, E.K. , and Poole, P.S. (2013) The plant microbiome. Genome Biol 14: 209. 23805896 10.1186/gb-2013-14-6-209 PMC3706808

[mbt213947-bib-0030] Van Peer, R. , Niemann, G.J. , and Schippers, B. (1991) Induced resistance and phytoalexin accumulation in biological control of Fusarium wilt of carnation by *Pseudomonas* sp. strain WCS417r. Phytopathology 81: 728–734.

[mbt213947-bib-0031] Voges, M.J.E.E.E. , Bai, Y. , Schulze‐Lefert, P. , and Sattely, E.S. (2019) Plant‐derived coumarins shape the composition of an Arabidopsis synthetic root microbiome. Proc Natl Acad Sci USA 116: 12558–12565. 31152139 10.1073/pnas.1820691116 PMC6589675

[mbt213947-bib-0032] Wagner, M.R. , Tang, C. , Salvato, F. , Clouse, K.M. , Bartlett, A. , Vintila, S. , *et al*. (2021) Microbe‐dependent heterosis in maize. Proc Natl Acad Sci USA 118: e2021965118. 34285069 10.1073/pnas.2021965118 PMC8325155

[mbt213947-bib-0033] Wei, G. , Kloepper, J.W. , and Tuzun, S. (1991) Induction of systemic resistance of cucumber to *Colletotrichum* *orbiculare* by select strains of plant growth‐promoting rhizobacteria. Phytopathology 81: 1508–1512.

[mbt213947-bib-0034] Yu, K. , Liu, Y. , Tichelaar, R. , Savant, N. , Lagendijk, E. , Van Kuijk, S.J.L. , *et al*. (2019a) Rhizosphere‐associated Pseudomonas suppress local root immune responses by gluconic acid‐mediated lowering of environmental pH. Curr Biol 29: 3913–3920. 31668625 10.1016/j.cub.2019.09.015

[mbt213947-bib-0035] Yu, K. , Pieterse, C.M.J. , Bakker, P.A.H.M. , and Berendsen, R.L. (2019b) Beneficial microbes going underground of root immunity. Plant Cell Environ 42: 2860–2870. 31353481 10.1111/pce.13632 PMC6851990

[mbt213947-bib-0036] Yu, K. , Stringlis, I.A. , Van Bentum, S. , De Jonge, R. , Snoek, B.L. , Pieterse, C.M.J. , *et al*. (2021) Transcriptome signatures in Pseudomonas simiae WCS417 shed light on role of root‐secreted coumarins in Arabidopsis‐mutualist communication. Microorganisms 9: 575. 33799825 10.3390/microorganisms9030575 PMC8000642

[mbt213947-bib-0037] Yuan, J. , Zhao, J. , Wen, T. , Zhao, M. , Li, R. , Goossens, P. , *et al*. (2018) Root exudates drive the soil‐borne legacy of aboveground pathogen infection. Microbiome 6: 156. 30208962 10.1186/s40168-018-0537-x PMC6136170

